# Immunotherapies of retinoblastoma: Effective methods for preserving vision in the future

**DOI:** 10.3389/fonc.2022.949193

**Published:** 2022-09-05

**Authors:** Ling Wang, Shixu Li, Jun Mei, Lin Ye

**Affiliations:** Shenzhen Eye Hospital, Jinan University, Shenzhen Eye Institute, Shenzhen, China

**Keywords:** retinoblastoma, immunotherapy, CART, bispecific antibody, monoclonal antibody

## Abstract

Retinoblastoma is the most common intraocular tumor in children. Patients can be cured by enucleation, but it can lead to vision loss. Chemotherapy is the main method of treatment for RB currently. Unfortunately, chemoresistant and tumor metastasis often happen, resulting in a relatively poor prognosis. Therefore, immunotherapy becomes one of the optimal choices. Targeting not only tumor cells but also the active tumor microenvironment is a novel strategy for RB treatment. Here, we conclude several potential targets for RB immunotherapy, including gangliosides GD2, PD-1 and PD-L1, B7H3, EpCAM and SYK. We also review the techniques for CART, bispecific antibodies and genetically modified Dendritic cells according to the characteristics of different targets and discuss the feasibility of immunotherapy with different targets.

## Introduction

Retinoblastoma has significant morbidity in young children and is one of the most common ocular tumors in children. Virtually all cases of retinoblastoma occur because of germline cancer susceptibility (1). Patients with this predisposition were also more likely to develop bilateral retinoblastoma. In children with this inherited disorder, retinoblastoma affects both eyes (bilateral) in 80% of cases and intracranial tumors (trilaterally) in 5%. Enucleation can cure children with unilateral intraocular retinoblastoma without any further treatment and subsequent vision loss. In localized tumor cases, and where appropriate, it can also be treated by laser application of cryotherapy or brachytherapy and/or local intra-arterial chemotherapy to save vision and preserve the eye. At present, the most common treatment for retinoblastoma remains systemic, subconjunctival, intraarterial, or intravitreal chemotherapy (2). It is also the current standard of care for managing orbital exenteration cases. The tumor unresponsiveness and recurrence are the most significant concern after chemo reduction. For larger tumors, systemic chemotherapy can achieve an initial decrease in tumor size, allowing for subsequent local treatment options. For unresponsive extraocular and/or metastatic disease, reserve high-dose systemic chemotherapy with stem cell rescue (3, 4). Despite high overall survival rates (> 95%) in Western countries, long-term survival is reduced in children treated with eye-preserving radiotherapy and/or chemotherapy compared with enucleation alone because of the higher incidence of secondary malignancies (5, 6). Retinoblastoma can be transmitted to the central nervous system *via* the optic nerve and to distant metastatic sites in lymph nodes, bone, bone marrow, and liver *via* the sclera *via* lymphatic or blood circulation to orbital bones. High-dose chemotherapy is often unsuccessful in rescuing in these cases, and because of its very aggressive nature, high-dose may cause lifelong sequelae to the patient (2, 7–9). As a local treatment modality, ophthalmic artery chemosurgery significantly reduced the rate of enucleation in unilateral and bilateral retinoblastoma, saving the majority of affected eyes without compromising survival. Although treatment outcomes are excellent in developed countries because of early diagnosis, patients with both metastatic and recurrent disease are common in developing countries, resulting in relatively poor prognosis. Therefore, it is essential to find new treatment strategies that are more effective and tolerable to effectively control retinoblastoma and protect eyeball and children’s vision, especially with minimal short- and long-term side effects.

Paradigm advances in cancer therapy have been made in the past decades, targeting not only tumor cells but also the active tumor microenvironment (TME) (10). The changes in the tumor microenvironment and protein communication between primary retinoblastoma and chemo-reduced retinoblastoma have not been reported. Therefore, it is important to understand the contribution of immune checkpoint markers in the microenvironment of retinoblastoma tumors. The TME comprises malignant and non-malignant cells such as cytokine, growth factors, extracellular proteins, endothelial cells, fibroblasts, and inflammatory cells (7). Targeting the tumor microenvironment has great potential because new immunotherapy strategies may be involved in tumor progression and metastasis (10). Despite the evolving nature of chemotherapeutic agents and the delivery of the agents, the development of novel targeted treatments requires a better understanding of the pathophysiology of retinoblastoma (9). Targeting the tumor microenvironment is less likely to cause adaptive mutations and metastasis because non-malignant cells are genetically more stable than tumor cells (11). Exploring functional changes in TME may provide essential considerations for ongoing studies of primary and chemo-reduced retinoblastoma. The use of immune checkpoint inhibitors has improved overall survival rates in treating many different solid tumors.

In the present review, we described the latest innovations in retinoblastoma immunotherapy targeting GD2, PD-1, B7H3, EpCAM and SYK.

## GD2

**Figure 1:**
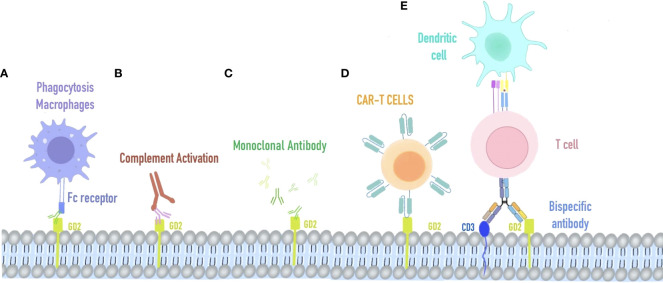
Immunotherapy strategy targeting GD2. **(A)** Macrophage phagocytosis combined with retinoblastoma cells mediated by anti-GD2 Fc receptors. **(B)** Complement activation on GD2-expressing retinoblastoma. **(C)** AntiGD2 monoclonal antibody is used for high-risk retinoblastoma. **(D)** CAR-T cells recognize retinoblastoma cells via their specific T cell receptors against GD2. **(E)** Bispecific antibody of GD2 and CD3, redirecting T cells and accessory immune cells (via their functioning Fc-fragment) toward retinoblastoma cells.

GD2, a disialoganglioside highly expressed in cancer cells (12), is involved in many signaling cascade pathways, such as MAPK, PI3K/Akt, and FAK/paxillin (13–15), in which cells can accelerate proliferation, migration, and stemness chemoresistance. Previous studies have focused on the diagnostic study of GD2 in some disseminated diseases such as bone marrow and cerebrospinal fluid. Since 1993, researchers have begun to examine the expression of GD2 and GD2 synthase in retinoblastoma (16). The most significant proportion of GD2 staining was studied in non-white populations. GD2 was expressed primarily on the membranes of retinoblastoma cells, and the positive rate of the assay was 37%, which suggests that GD2 has the capacity to be a potential therapeutic target for RB (17, 18). The heterogeneous expression of GD2 in positively stained samples further demonstrates a multifocal origin and distinct cytogenetic clones within a tumor (19). The relationship between GD2 expression and tumor stage and proliferation index suggests that GD2 expression is associated with poor patient prognosis (20). GD2 is widely expressed in retinoblastoma, and MYCN amplification in pretreated chemo-refractory cases, suggesting that for treatment of RB, anti-GD2 monoclonal antibodies may be effective (21, 22). Anti-GD2 mAbs have three proposed mechanisms of action against GD2-expressing tumor cells. First, GD2 mAbs initiate the phagocytosis by macrophages (**Figure 1A**) destruction of tumor cells by natural killer cells and the cytotoxicity of granulocytes mediated by killing tumor cells. Second, GD2 mAbs mediate the lysis of tumor cells *via* complement-dependent cytotoxicity (**Figure 1B**). Third, GD2 mAbs direct induction of cell death due to the specific binding of anti-GD2 mAbs to GD2 (**Figure 1C**) (23). In Michelle’s study (24), intending to improve survival in high-risk neuroblastoma, researchers used an anti-GD2-based monoclonal antibody (dinutuximab) in the maintenance phase of treatment. COG-ANBL0032 protocol comparing the ch14.18 antibody (dinutuximab) in combination with isotretinoin and alternating GM-CSF and IL-2 to single-agent isotretinoin in the maintenance phase of treatment. There were 20% and 11% increases in event-free survival (EFS) and 11% and 16% increases in overall survival (OS) after 2 and 5 years, respectively.

Improved early response and outcome of GD2 monoclonal antibody (hu14.18K322A) in children with newly diagnosed high-risk neuroblastoma by six cycles of concurrent induction chemotherapy with hu14.18K322A, GM-CSF, and low-dose IL-2 was evaluated by another group. After the first two cycles of chemoimmunotherapy, 42 of 63 evaluable patients had partial responses (PRs) or better. At the end of induction, partial responses or better were seen in 60 of 62 patients (97%). No patient developed progressive disease throughout the induction period (25).After being tested in clinical trials, anti-GD2 monoclonal antibodies proved their safety and efficacy suggesting that GD2 could be an essential immune target for the treatment of RB (21).

T lymphocytes isolated from patients were designed to express CD19-specific chimeric antigen receptors (CARs) and showed significant antitumor effects against acute B-cell leukemia and non-Hodgkin’s lymphoma. CAR-T has two distinguishing features: substantial toxicity of cytotoxic T lymphocytes and specific antigen-binding of monoclonal antibodies. It led to the creation of a GD2-specific chimeric antigen receptor (CAR)-modified T-cell therapy for retinoblastoma (**Figure 1D**) Sujjitjoon et al. developed a novel 4SCAR-GD2 T for the treatment of retinoblastoma (26). The intracellular domain of 4SCAR-GD2 T contains CD28, 41BB, and CD3ζ, and its scFv fragment derived from the monoclonal antibody hu3F8, recognizes human GD2 (**Figure 1E**) *In vitro* studies using Y79RB cells found that this 4SCAR-GD2 T had high cytotoxicity. To mimic the high tumor burden *in vivo*, the investigators increased the number of Y79RB cells by 3-fold after the first round of killing and prolonged the co-culture time of 4SCAR-GD2 with Y79RB. After 6 days of co-culture, some Y79RB cells survived with reduced expression of GD2 on their cell surface compared to before (from 93.2% to 65.5%). Typically, immune checkpoint blockade is the main reason for tumor cell escape. Therefore, further detection was carried out and revealed that there was no PD-L1 expression on the surface of parental Y79RB cells. After co-culture with 4SCAR-GD2 T, the expression of PD-L1 on the surface of Y79RB cells was up-regulated, and the expression of PD-1 on the surface of 4SCAR-GD2 T in the co-culture system was also up-regulated. This result indicates that PD1: PD-L1 is involved in the immune escape of tumor cells and suppresses the function of CAR T cells after repeated antigen exposure.

## PD-1

**Figure 2:**
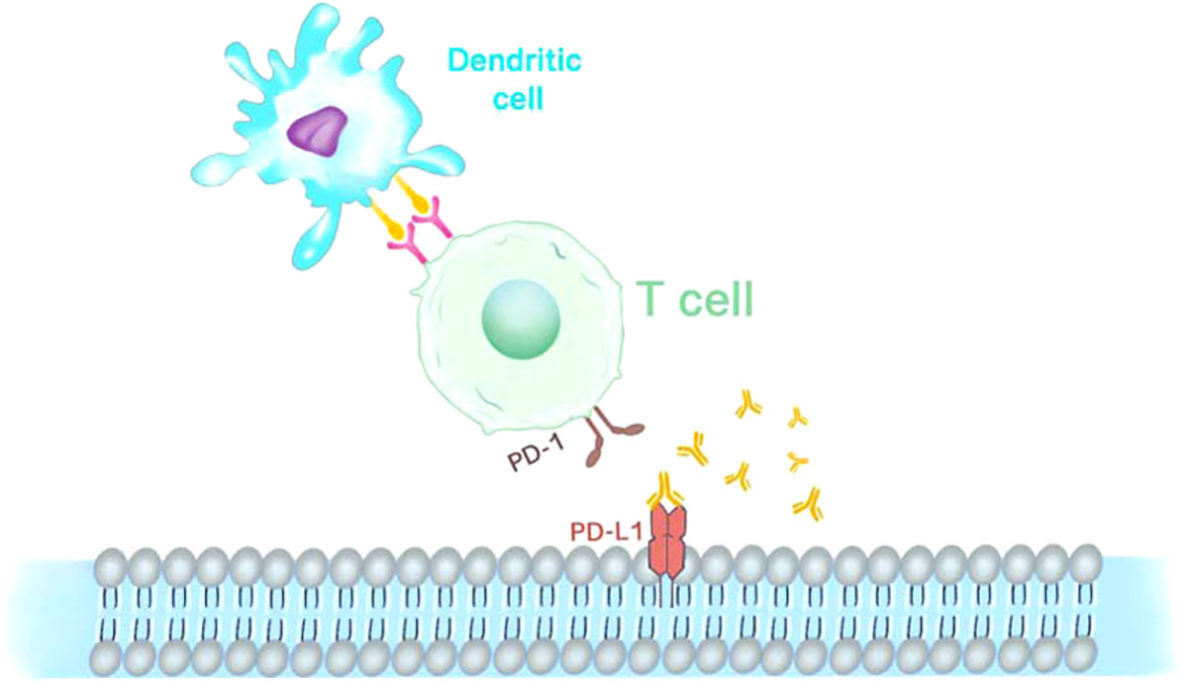
Immunotherapy anti PD-1 and PD-L1. PD-1 is expressed on the surface of CAR-T cells as an inhibitory receptor, while its ligands PD-L1 is mainly expressed in antigen-presenting cells and tumor cells.

Programmed cell death 1 (PD-1), programmed cell death ligand 1 (PD-L1), and cytotoxic T lymphocyte-associated antigen-4 (CTLA-4), have been the focus of research in immunotherapy fields (**Figure 2**). Promising results regarding their efficacy in fighting tumors in patients with advanced tumors continue to emerge (27). An indication of cancer, as noted in numerous publications, is the absence of immune control (28). Closely associated with tumorigenesis and progression and playing a key role in tumor immune escape and TME formation is PD-1 and its ligand PD-L1 (29). PD-1 is commonly expressed on the surface of activated immune cells, such as T cells, B cells, and bone marrow cells. These two ligands, PD-L1 (B7-H1) and PD-L2 (B7-DC), expressed mainly in the placenta, tonsil, and retina, both belong to the B7 family of cell surface glycoproteins (30).PD-L1 is expressed in non-hematopoietic cells such as endothelial, epithelial, and tumor cells and appears in dendritic cells, myeloid cells, T and B cells, and other hematopoietic cells (31). A widely accepted method to assess PD-1/PD-L1 expression in cancer biology is immunohistochemistry. The most widely used practice for predictive biomarker detection of anti-PD-1/PD-L1 and CTLA-4 therapies in tumors is IHC for PD-L1 protein expression (32). The role of PD-1 in cancer immune evasion has been demonstrated because, as a ligand for PD-1, PD-L1 is highly expressed in some cancers (33). PD-L1 expression is not difficult to find in many tumor types, such as melanoma and glioblastoma, lung, kidney, head and neck, gastric, colon, pancreatic, breast, cervical, uterine, and ovarian cancers (34). PD-L1 is also expressed in hematological malignancies, such as multiple myeloma, lymphoma, and various leukemia types, and is associated with a worsening prognosis (35). However, the altered pattern of tumor microenvironment in primary and chemotherapeutic tumors has been documented in previous studies. However, the differences in histopathological findings and expression of immune markers in cases of primary retinoblastoma (group I) and chemotherapeutic retinoblastoma (group II) have remained to be studied to date. In Singh’s study, the expression patterns of PD-1, PD-L1, and CTLA-4 proteins differed in both groups of retinoblastomas. There was increased expression of PD-L1 (46/144) and decreased expression of PD-1 (29/144) in primary retinoblastoma. A statistically significant overall survival rate was observed in PD-L1-expressing tumors (89.13%; P value = 0.015) compared to PD-1 expression (93.10%; P value = 0.394). In chemically induced retinoblastoma, on the other hand, the opposite pattern was observed, with increased expression of PD-1 (48/118) and decreased expression of PD-L1 (22/118). PD-1 expression was statistically found to correlate with overall survival in chemically induced patients (63.28%; P value = 0.003). While no clear correlation was found with patient outcomes, CTLA-4 protein expression revealed a similar pattern in both primary and chemically induced retinoblastoma. While evidence suggests that intrinsic expression of PD-1 promotes tumor growth independent of adaptive immunity in a variety of factors involving gene copy number alterations, epigenetic modifications, and the tumor microenvironment in tumor cell lines, the exact mechanism by which PD-1 may be expressed within tumor cells has not been clarified (32, 36–38).

## B7H3

PD-L1 and PD-1, members of the B7 family, have been evaluated in many studies for both expressions in RB (39, 40). Researchers previously subjected primary retinoblastoma and retinal tissue to a membrane proteomics study (41). The study compared their expression of immunotherapeutic molecules, and one of the B7 family checkpoint molecules, B7-H3 (CD276), was overexpressed in RB tumors compared to retinal tissue. Many studies have shown that overexpression of B7H3 in some malignancies can cause metastasis or severe complications of cancer (42–47). B7H3 expression is highly heterogeneous. Interestingly, when B7H3 levels are high in the lobules, they are deficient in the blood vessels in the areas adjacent to the lobules and vice versa. Several studies have reported the expression of B7H3 in tumor vessels and tumor cells (48). In different diseases, B7H3 is differentially expressed in the stroma and tumor cells; for example, in colorectal and pancreatic cancers, a higher percentage of B7H3 was positive in stroma than in tumor cells, whereas in prostate cancer, B7H3 expression was higher in tumor cells than in stroma; in RB tumors, B7H3 was observed in mutually exclusive expression in tumors and blood vessels, which has not been reported in other cancer types. This result needs to be further investigated and examined whether it is related to cells in the vasculature, such as endothelial cells, or stromal cells surrounding the vasculature, such as pericytes and fibroblasts, or whether it is related to differences between pre-existing and newly generated vessels. Since the clinical importance of any target molecule in RB tumors depends on certain histopathological features, the expression of B7H3 in terms of differentiation status, site of invasion, and degree of asexual reproduction of the tumor which is closely related to the prognosis of the disease were investigated. Among these, in terms of differentiation status, B7H3 is highly expressed in poorly differentiated RB and less expressed in moderately or well-differentiated RB tumors. A retrospective study of 326 primary RB tumors, showed that poorly differentiated tumors were significantly associated with more than three high-risk symptoms, particularly massive choroidal invasion (49). The high expression of B7H3 in such tumors is beneficial for targeted therapy.

The common metastatic areas of RB tumors are the central nervous system (CNS), regional lymph nodes, bone marrow, and bone (50). Its invasive status determines the areas where it metastasizes. The invasion sites are classified as neurological and non-neurological, depending on their prognosis and the area of metastasis. Neurological invasion leads mainly to CNS metastasis, whereas non-neurological invasion tends to metastasize more to other systemic sites (51). Among them, CNS metastasis has a poorer prognosis, probably because chemotherapeutic agents cannot cross the blood-brain barrier19, in which case adjuvant intrathecal or intracerebral chemotherapy is required (52, 53). Compared with neural tissue, B7H3 expression in invading non-neural tissue of RB tumors showed a significant increase (54). B7H3 expression may be suppressed when the tumor invades the optic nerve (40). However, we could not find any support from the published literature. One limitation is the number of samples that could be analyzed for this correlation; however, if built with a larger cohort, this finding may have clinical implications for the use of B7H3 as a therapeutic approach.

**Figure 3:**
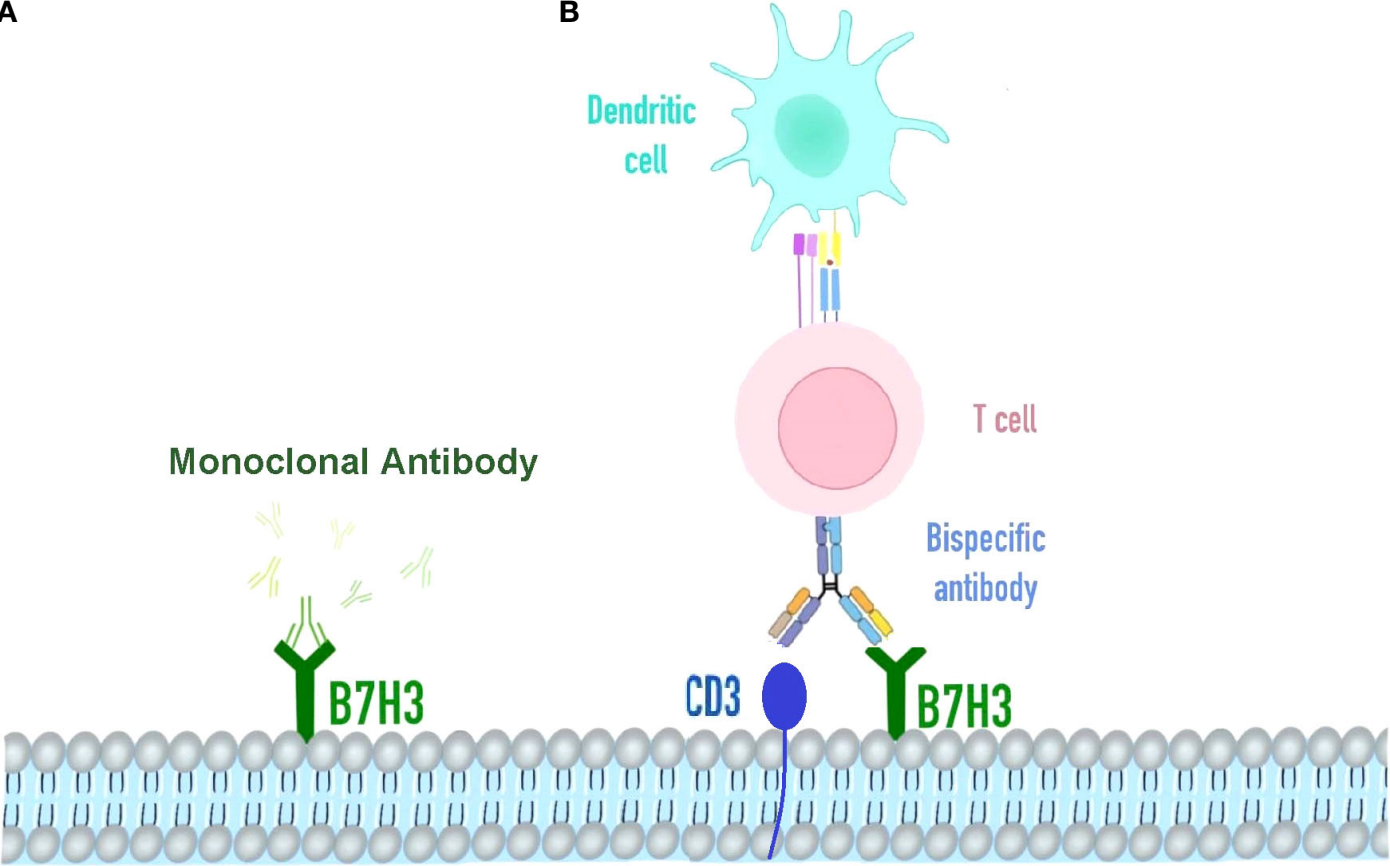
Immunotherapy strategy targeting B7H3. **(A)** Omburtamab is a radionuclide iodine-131-labeled monoclonal antibody targeting B7H3 cells in various solid tumors, including retinoblastoma. It binds to the FG cyclically dependent conformation, a key region of the biological function of the B7-H3 molecule. **(B)** Bispecific antibody of B7H3 and CD3, redirecting T cells and accessory immune cells.

There is a significant anti-tumor activity demonstrated by B7-H3-targeted CAR-T cells against AML and melanoma for both *in vitro* and xenograft mouse models. In clinical trials, multiple therapeutic agents targeting B7-H3 have been conducted. As an Fc-optimized monoclonal antibody (mAb) against B7-H3, Enoblituzumab has been evaluated together with an anti-programmed death 1 (PD-1) monoclonal antibody in patients with B7-H3-expressing solid tumors during phase I clinical study (**Figure 3A**). Another B7-H3-targeting antibody for the treatment of brain and central nervous system tumors, neuroblastoma, and carcinoma, radiolabeled 8H9, was also evaluated in a phase I trial. (ClinicalTrials.gov: NCT00089245). MGD009 is a bispecific antibody developed by MacroGenics against B7-H3 and CD3, while the FDA partially shelved the two clinical studies on MGD009 due to hepatotoxic events in monotherapy trials, such as reversible transaminase level increases with or without concomitant bilirubin level increases (55) (**Figure 3B**).

The presence of B7H3 in RB tumors opens the way for developing targeted therapeutic and immunotherapeutic approaches. Furthermore, it is interesting to observe that the expression of B7H3 is reduced when the tumor enters the optic nerve, so the next step should focus on the presence of molecules that reduce B7H3 in the optic nerve bundle and their implications for clinical treatment. Clinical data with 1 to 4 years of follow-up did not show any significant correlation between patient survival and B7H3 expression. One with long-term follow-up data is needed further to understand the correlation between B7H3 expression and RB prognosis.

## EpCAM

**Figure 4:**
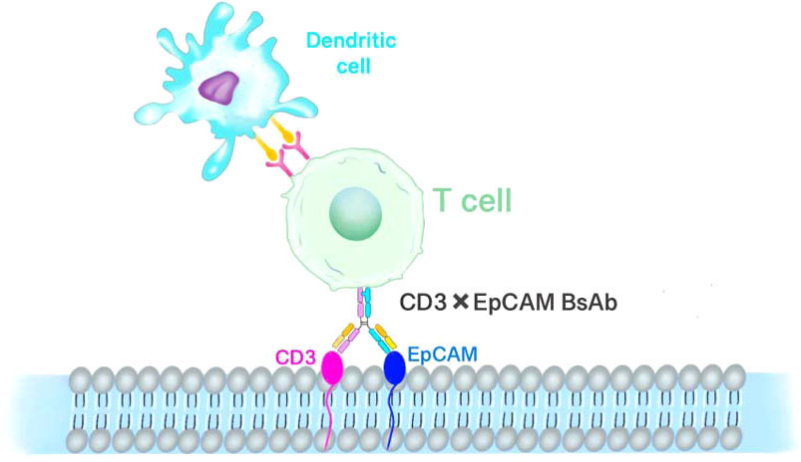
T-cell-mediated immunotherapy of EpCAM. EpCAM and CD3 bispecific antibodies redirect T lymphocytes to attack retinoblastoma cells.

Epithelial cell adhesion molecule (EpCAM) was earlier considered as a marker for adult liver stem/progenitor cells and oval cells (56, 57), which is an epithelial cell adhesion molecule with all the characteristics of tumor stem cells (CSCs). EpCAM is highly expressed in aggressive tumors compared to RB, a non-invasive tumor. Damages to the EpCAM gene may result in a substantial decrease in cell proliferative capacity (58). Bispecific antibodies (bsAb) are artificial molecules with dual specificity for two separate antigens. The most common bsAb antigen on lymphocytes is an invariant CD3 signaling complex that induces the activation of polyclonal T cells. A number of anti-EpCAM bsAb and single-chain antibodies have been produced and tested as immunotherapeutics (**Figure 4**) (56, 59–62). The host antitumor immunity has a significant contribution to preventing the development of malignant tumors. However, when tumor cells lack tumor-associated antigens or various co-stimulatory or major histocompatibility complex molecules, the host mononuclear cells may become dysfunctional. Aggressive RB primary tumors express low levels of human leukocyte antigen (HLA) class I and II antigens, which may be an advantage for tumor cells to escape t-cell or natural killer (NK) cell-mediated attack (63). In this context, the potential of a novel therapeutic modality using the bispecific antibody-directed T-cell attack on tumor cells may become a promising treatment for retinoblastoma. The bispecific antibody can effectively induce lysis of tumor cells *in vitro*, thus reducing the production of malignant ascites in patients with advanced ovarian cancer (64). Mitra et al. studied the role and expression of EpCAM in the development of retinoblastoma (65). The study found that EpCAM+Y79 cells have strong proliferation and invasion ability and neurosphere formation ability. Using fresh retinoblastoma tissue, the co-expression of EpCAM and three other putative tumor stem cell markers CD44, CD24 and ABCG2 was examined. The results showed that not every tumor tissue expressed CD44, CD24 or ABCG2, but the expression of EpCAM could be detected. The use of preactivated PBMC and bispecific antibodies to EpCAM × CD3 can promote lysis of RB cells. Therefore, targeting CSC combined with conventional chemotherapy should be the basic therapeutic strategy for eradicating tumors. EpCAM is an attractive target for bsAb and bispecific single-chain antibodies for antitumor therapy (66–68).

Moreover, EpCAM×CD3 activity is dose-dependent and increases within 24 h. This effect was consistently observed in all five tumor types examined. The production of effector cytokines was raised in the supernatant of cultures containing EpCAM+ cells and pre-activated PBMC as well as EpCAM×CD3, as demonstrated by our ELISA assay. In summary, EpCAM×CD3 potently stimulates the secretion of effector cytokines by pre-activated lymphocytes in the presence of EpCAM-expressing tumor cells. Activated T cells secreting TNF-α, IFN-γ, and chemokines may increase efficacy by enhancing immune cell attraction and stimulation. It has been proven that high levels of IL-10 in the tumor microenvironment facilitate tumor rejection by potentiating the cytotoxicity of T lymphocytes (69). TGF-β functions as a tumor cell suppressor (70), suggesting that a bispecific antibody-mediated immunotherapeutic approach may potentially help manage the proliferation of RB tumor cells. A high percentage of cells in retinoblastoma express EpCAM, and especially tumors with optic nerve/choroidal invasion demonstrate increased EpCAM expression (63). Therefore, the invasive retinoblastoma is an attractive tumor for therapeutic targeting using a bispecific antibody (EpCAM × CD3). In summary, EpCAM + RB cells behave *in vitro* similarly to tumor stem cells. In the presence of EpCAM expressing RB tumor cells, EpCAM×CD3 has potent anti-tumor activation *in vitro via* induction of interleukin and cytokine secretion by pre-activated lymphocytes.

## SYK

The spleen tyrosine kinase (SYK) is one of the most dramatically upregulated kinase genes in RB cells (71). It is involved in signaling the inflammatory cell B-cell receptor complex in the inflammatory response and has also been associated with hematopoietic malignancies (72–74). There are two SYK isoforms in tumor cells, the full-length SYK (SYK-L) and the variable splice SYK transcript (SYK-S). Among them, SYK-L can enter the nucleus and prevent cancer cell invasion. At the same time, SYK-S is only found in the cytoplasm, where it can promote tumor development and is a proto-oncogene involved in the survival of RB cells. However, SYK is not expressed in retinal progenitor cells or neurons, and no function has been found in the developing visual system. ChIP-on-chip analysis revealed increased histone activation modifications (H3K4me3 and K3K9/14Ac) at the SYK promoter, whereas the histone repression marker (H3k9me3) was unchanged in human retinoblastoma *in situ* xenografts and cell lines (71). There was also an increase in RNA polymerase II bound to the SYK promoter. ChIP-on-chip results confirmed increased expression of the SYK gene. SYK protein was found at higher levels in human retinoblastoma *in situ* xenografts and cell lines than in human fetal retina. Retinoblastoma tissue microarrays (TMA) or whole eye sections were subjected to immunohistochemistry. The results indicated that SYK was heavily expressed (3+) in all tumor cells (82/82), while normal retinas had no expression of SYK. The kinase activity of SYK is regulated by autophosphorylation of the Tyr525/526 residues within its catalytic domain. In retinoblastoma cells, the sites are phosphorylated and reversed.

Although SYK was consistently immunonegative in non-neoplastic lesions and pseudo retinoblastoma eyes, conversely, it was histologically immunopositive in any RB eyes (75). Strong immunostaining of SYK is found in RB eyes - the nucleus and cytoplasm of RB cells. While SYK is silenced in benign retinas, it is activated in RB. In differentiating malignant tumors from benign diseases in the retina, SYK is also a good marker. SYK, an important promoter of tumorigenesis in RB, showed a more significant negative correlation between its expression and tumor necrosis. However, pseudo retinoblastoma is usually undetectable by clinical and diagnostic imaging techniques because its symptoms and clinical findings are comparable to those of RB. The above results suggest that SYK can be used in a protein-based or genetic approach to differentiate these disease possibilities and so is a useful clinical marker.

**Figure 5:**
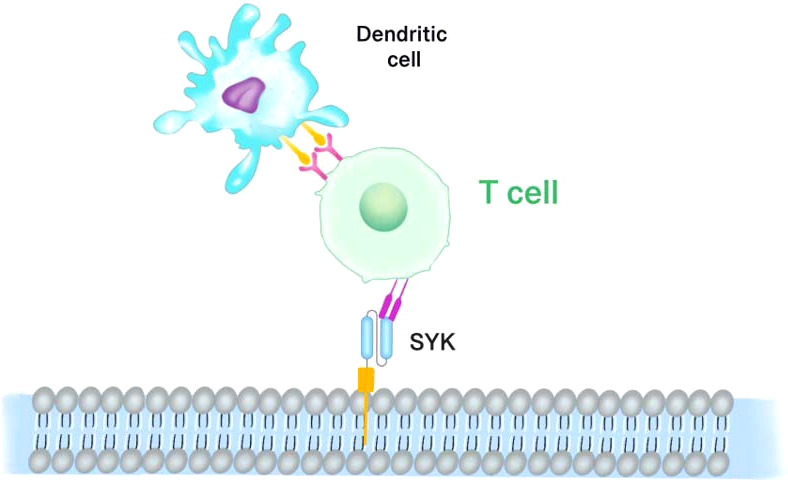
SYK-targeted dendritic cell-mediated CAR-T cells. Dendritic cells (DCs) which expressing and presenting the SYK peptide antigen are modified to cytotoxic T lymphocytes (CTL). SYK-overexpressing DCs induce the cytotoxicity of CTL.

Since SYK expression is required for retinoblastoma growth and survival, X Chen et al. (76) synthesized SYK shRNA and cloned it into the lentivirus vector Lenti-SYK-9. In addition to accelerating apoptosis of retinoblastoma cells, Lenti-SYK-9 effectively removed SYK from retinoblastoma cell lines. Further to the previous efforts, the researchers used lentivirus to genetically modify dendritic cells (DC) to make cytotoxic T lymphocytes (CTL) express SYK antigens *in vitro*. SYK-negative cell lines (MDA-MB-231, MCF-10A, hTERT-RPE1) and SYK-positive cell lines (MCF-7 and RB-Y79) were used to assess the specificity and cytotoxicity of DCs expressing CTLs. The CTL toxicity triggered by SYK-high expression in DCs **Figure 5** (SYK-DC-CTLs) elevated the killing effect on SYK-positive cells by more than three times compared to SYK-negative cells. SYK-modified DCS had a CTL cytotoxic effect on SYK-positive cell lines, but no killing effect on SYK-negative cell lines. Although SYK-silenced RB-Y79 cells potently bypassed the cytotoxic attack of SYK-DC-CTL, SYK-DC-CTLs were overexpressed in hTERT-RPE 1 cells, suggesting that SYK is a specific antigen for Rb. In addition, SYK-DC-CTL had specific cytotoxic effects on carboplatin-resistant RB-Y79 cells *in vitro*.

Exposure of Y79 cells to different doses of lymphocyte-derived microparticles (LMPs) was studied by Qian Q et al. (77). The results revealed that SYK mRNA was significantly diminished with 20 μg/ml of LMPs. For 24 h, treatment of human retinoblastoma cells with 20μg/ml LMPs was carried out and the expression of SYK protein was analyzed by Western blot. The results also showed that LMPs significantly inhibited the expression of SYK protein. LMPs can downregulate SYK and induce retinoblastoma cell death, as further supported by the immunohistochemical results of SYK expression.

These findings suggest that this gene may contribute to RB tumor development (71), and therefore SYK may be a potential target for RB therapy.

## Discussion

The World Health Organization (WHO) has selected Retinoblastoma as a high-priority tumor for the Global Initiative for Childhood Cancer. The initial cure rate is high, yet it is potentially lethal when not treated promptly. Ocular palliative approaches have made great strides during the last few decades, making it the most treatable pediatric cancer for intraocular retinoblastoma in a high-income country. There have been developments in delivery methods locally enabling chemotherapy to maximize exposure in the retinal, subretinal, and vitreous spaces, i.e., improved techniques for safe ophthalmic artery chemosurgery (OAC) and intravitreous chemotherapy (IVi) injections which have led to the maintenance of ocular and visual acuity at levels never seen before. Critically, these new local therapies provide the retina and optic nerve access to chemotherapy at very high intensities, as seen in preclinical models, thus capable of blocking the spread of tumors to the central nervous system. After more than a decade of consistent access in major clinical centers worldwide and over 200 publications related to this field, the OAC and IVi have proven safe and reliable without increasing the risk of metastatic dissemination. By eliminating EBRT and systemic chemotherapy, long-term survival is improved with these therapies by reducing the incidence of treatment-related severe toxicities, the risk of secondary malignancies, and associated mortality.

Unfortunately, children suffering from disseminated retinoblastoma have virtually no options for treatment. New therapeutic strategies are expected to be highly effective for both intraocular and extraocular diseases, provided that the risk of toxicity is lower. In addition, the availability of more new non-chemotherapy therapies gives patients more options, such as targeted therapies, immunotherapy, and lysing viruses.

## Author contributions

LW, JM and LY contributed to the conception of the manuscript. LW and SL wrote the manuscript and drew the pattern diagrams. All authors contributed to the article and approved the submitted version.

## Funding

This study was funded by the National Natural Science Foundation of China (81870626), Science and Technology Plan Project of Shenzhen, China (JCYJ20170306123423907, JCYJ20210324125805012).

## Conflict of interest

The authors declare that the research was conducted in the absence of any commercial or financial relationships that could be construed as a potential conflict of interest.

## Publisher’s note

All claims expressed in this article are solely those of the authors and do not necessarily represent those of their affiliated organizations, or those of the publisher, the editors and the reviewers. Any product that may be evaluated in this article, or claim that may be made by its manufacturer, is not guaranteed or endorsed by the publisher.
